# Critical Aspects of Metal–Organic Framework‐Based Materials for Solar‐Driven CO_2_ Reduction into Valuable Fuels

**DOI:** 10.1002/gch2.202000082

**Published:** 2020-11-25

**Authors:** Yiqiang He, Chunguang Li, Xiao‐Bo Chen, Heng Rao, Zhan Shi, Shouhua Feng

**Affiliations:** ^1^ State Key Laboratory of Inorganic Synthesis and Preparative Chemistry Jilin University Changchun 130012 P. R. China; ^2^ School of Engineering RMIT University Carlton VIC 3053 Australia; ^3^ International Center of Future Science Jilin University Changchun 130012 P. R. China

**Keywords:** CO _2_ reduction, fuels, photocatalysis, porous materials, solar energy

## Abstract

Photoreduction of CO_2_ into value‐added fuels is one of the most promising strategies for tackling the energy crisis and mitigating the “greenhouse effect.” Recently, metal–organic frameworks (MOFs) have been widely investigated in the field of CO_2_ photoreduction owing to their high CO_2_ uptake and adjustable functional groups. The fundamental factors and state‐of‐the‐art advancements in MOFs for photocatalytic CO_2_ reduction are summarized from the critical perspectives of light absorption, carrier dynamics, adsorption/activation, and reaction on the surface of photocatalysts, which are the three main critical aspects for CO_2_ photoreduction and determine the overall photocatalytic efficiency. In view of the merits of porous materials, recent progress of three other types of porous materials are also briefly summarized, namely zeolite‐based, covalent–organic frameworks based (COFs‐based), and porous semiconductor or organic polymer based photocatalysts. The remarkable performance of these porous materials for solar‐driven CO_2_ reduction systems is highlighted. Finally, challenges and opportunities of porous materials for photocatalytic CO_2_ reduction are presented, aiming to provide a new viewpoint for improving the overall photocatalytic CO_2_ reduction efficiency with porous materials.

## Graphical abstract

Porous materials, including metal–organic frameworks, covalent organic frameworks, zeolites, and inorganic/organic porous semiconductors used for photocatalytic CO_2_ reduction are reviewed. Porous materials have been intensively exploited in solar‐driven CO_2_ reduction reactions for making valuable fuels. Three critical aspects of CO_2_ photoreduction are summarized and discussed, which provide new guidance for the future practical applications of porous materials.

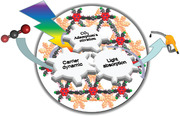

## 1 Introduction

**Figure 1:**
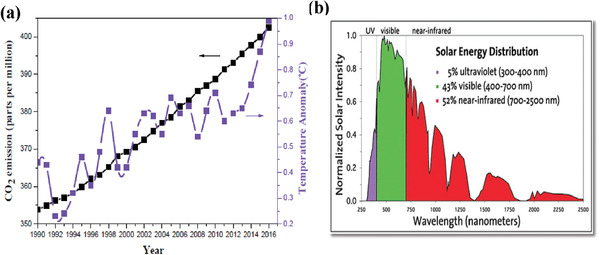
a) Annual CO_2_ emission in the atmosphere and global mean surface temperature as a function of years from 1990 to 2016.^[^ ^1^ ^]^ b) UV–vis–IR sunlight spectrum. Reproduced with permission.^[^ ^9^ ^]^ Copyright 2019, Wiley‐VCH.

With excess consumption of fossil fuels, as presented in **Figure** **1**a, the annual carbon dioxide (CO_2_) emission and global surface temperature are ever‐increasing rapidly, which indicates that the “greenhouse effect” will increase profoundly if such a consumption of fossil fuels continues.^[^ ^1^, ^2^, ^3^ ^]^ There are two common strategies to mitigate the “greenhouse effect” through decreasing the concentration of CO_2_ in the atmosphere. One solution is sequestering CO_2_ in geological media through physical or chemical absorption. The other one is direct conversion of CO_2_ into valuable fuels, such as CO, HCOOH, methanol, methane, ethane, etc. Such an economic pathway can not only reduce the atmospheric CO_2_ concentration, but also provide significant chemical energy as substitutions to fossil fuels.^[^ ^4^, ^5^ ^]^ Up to now, several energy sources with a large diversity of technical routes have been proposed to convert CO_2_ into fuels, including thermochemical reduction, photochemical reduction, electrochemical reduction and biochemical reduction.^[^ ^6^, ^7^ ^]^ Of those, solar energy outweighs the others given its pollution‐free, inexhaustible, and cost‐free nature.^[^ ^8^ ^]^ Hence, conversion of solar energy into chemical energy through photocatalytic CO_2_ reduction is worth of investigating.

A typical process of CO_2_ photoreduction involves three critical aspects. 1) Light absorption. Ultraviolet (UV) light accommodates high energy which readily excite photocatalysts, however, it only accounts for about 5% of the entire solar irradiance. Although inferred (IR) light accounts for more than a half, its low energy does not suffice to activate photocatalysts. Visible light, which accounts for about 43%, will be a sound and reliable source to be used for photocatalysis (Figure 1b).^[^ ^9^ ^]^ To obtain high solar energy conversion efficiency, a large number of visible‐light responsive semiconductor materials have been investigated.^[^ ^10^ ^]^ 2) Carrier dynamics include carrier separation, migration, trap, In general, light irradiation of a photocatalyst leads to the formation of photogenerated electron and hole pairs. Then they migrate toward the surface of the photocatalysts to participate in reduction and oxidation reactions, respectively.^[^ ^11^ ^]^ 3) Adsorption and activation of CO_2_ molecules and reaction on the surface. In this process, CO_2_ molecules are expected to be adsorbed on the surface of photocatalyst. Therefore, the capacity of uptake CO_2_ is the key. Concentration of the adsorbed CO_2_ on the surface of a photocatalyst and number of activated catalytic sites determine the efficiency of CO_2_ reduction process.^[^ ^12^ ^]^ In addition, some functional groups modified on the surface such as hydroxyl, amino, frustrated Lewis pairs (FLPs), could activate the adsorbed CO_2_ molecules and decrease the reaction energy barrier, which will boost the efficiency of CO_2_ reduction into fuels.^[^ ^13^, ^14^ ^]^ A large number of inorganic semiconductors have been developed as inspired by the pioneering work led by Fujishima and co‐workers in terms of employment of TiO_2_ for solar‐driven CO_2_ reduction in 1979.^[^ ^15^, ^16^ ^]^ In the recent years, significant advances have been achieved in expanding the visible‐light absorption range and facilitating solar energy conversion efficiency.^[^ ^17^, ^18^, ^19^, ^20^, ^21^, ^22^, ^23^ ^]^ However, the performance of inorganic semiconductors remains unsatisfactory with respect to industrial applications, such as low capacity of CO_2_ absorption, limited specific surface area, large bandgap, electron–hole recombination in nonporous^[^ ^24^ ^]^ and low photocatalytic CO_2_ reduction selectivity of high value‐added fuels. As such, it is of paramount significance to design and develop efficient and selective photocatalytic CO_2_ reduction systems with possession of extended visible‐light absorption ability, efficient photogenerated charge separation, excellent CO_2_ molecular adsorption capacity, and abundant active CO_2_ reduction sites.

In comparison with traditional porous/nonporous materials, metal–organic frameworks (MOFs), covalent–organic frameworks (COFs), and zeolites, recently have attracted considerable attention owing to low density, large surface area, high porosity, structural and compositional diversity, which holds great potential for a broad range of commercial aspects in physical, mechanical, acoustical, thermal, and electrical fields.^[^ ^25^, ^26^ ^]^ Benefiting from these specialties, porous materials have been widely applied in catalysts, sensors, gas adsorption and separation, drug delivery, and environmental governance.^[^ ^27^, ^28^, ^29^, ^30^, ^31^, ^32^, ^33^, ^34^, ^35^, ^36^, ^37^, ^38^ ^]^ In particular, solar‐driven CO_2_ reduction could be catalyzed by proper porous materials, owing to the tunable light absorption ability over broad range, the ameliorative carrier separation, the evenly distributed catalytic active site and the ideal catalytic platform for mechanism study of structure–activity relationships. In addition, the potential CO_2_ capture capability of porous materials further endows their merits toward photocatalytic reduction of CO_2_ into value‐added fuels by concentrating CO_2_ molecules at active sites.^[^ ^39^ ^]^

**Scheme 1:**
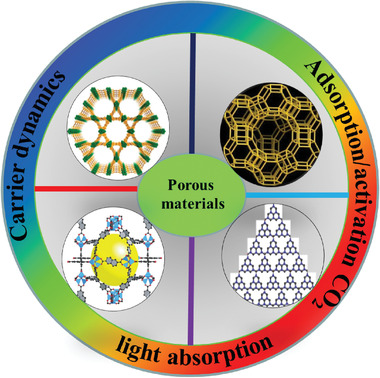
Photocatalytic CO_2_ reduction with porous materials.

To make a comprehensive understanding of rational design and development of more creative porous materials for solar‐driven CO_2_ reduction, it is necessary to provide a timely research progress report to capture the state‐of‐the‐art progress in the field. Several reviews have focused on MOFs‐based materials from the perspectives of categories of photocatalytic products, wide applications such as water splitting and cycloaddition of CO_2_.^[^ ^40^, ^41^, ^42^, ^43^, ^44^, ^45^, ^46^ ^]^ This report mainly summarized the state‐of‐the‐art progress of MOFs‐based photocatalytic systems for CO_2_ reduction; together with COFs‐based, zeolite‐based, inorganic porous semiconductors or organic polymers photocatalyst. In this report, we highlight representative approaches for optimizing the performance of CO_2_ photoreduction through using versatile porous materials and three main critical aspects will be discussed (**Scheme** **1**). It includes the present fundamental of CO_2_ photoreduction and the recent four types of photoactive porous materials applied in CO_2_ reduction. Finally, challenges and prospects of porous materials for photocatalytic CO_2_ reduction are illustrated.

## 2 Fundamentals of Porous Materials for CO_2_ Photoreduction

**Figure 2:**
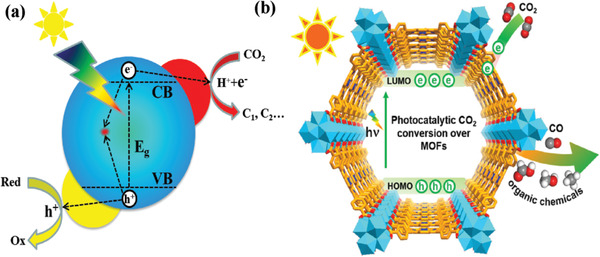
Schematic illustrations of a) photocatalysis over a semiconductor and b) photocatalytic CO_2_ reduction into organic chemicals over MOFs.^[^ ^49^ ^]^ Reproduced with permission.^[^ ^49^ ^]^ Copyright 2020, Elsevier.

Photoreduction of CO_2_ into chemical feedstocks is a promising solution to energy crisis and environmental problems. A typical process of photocatalysis includes three basic but critical principles. As shown in **Figure** **2**a, semiconductor adsorbs sunlight of energy ≥*E*_g_ (bandgap) and generates electron–hole pairs simultaneously. Then photogenerated electrons transfer from valence band (VB) to conduction band (CB), leading to the separation of electron–hole pairs. Subsequently, the excited electrons and holes are transferred to the surface to take part in reduction reaction and oxidation reaction process, respectively. It is noted that CB potential of a semiconductor must satisfy the thermodynamic potential of different products. The main potentials of a range of CO_2_ reduction products are listed in **Table** **1**, which are referred the normal hydrogen electrode (NHE) at pH 7, namely *E* ^0^ *V* versus NHE at pH 7.^[^ ^47^, ^48^, ^49^ ^]^

**Table 1:** Different products potentials with reference to NHE at pH 7

Products	Reaction	*E* ^0^ (V vs NHE)	Equation
CO_2_^−^	CO_2_ + e^−^ → CO_2_^·−^	−1.90	(1)
CO	CO_2_ + 2H^+^ + 2e^−^ → CO + H_2_O	−0.53	(2)
CH_4_	CO_2_ + 8H^+^ + 8e^−^ → CH_4_ + 2H_2_O	−0.24	(3)
CH_3_OH	CO_2_ + 6H^+^ + 6e^−^ → CH_3_OH + H_2_O	−0.38	(4)
HCHO	CO_2_ + 4H^+^ + 4e^−^ → HCHO + H_2_O	−0.48	(5)
HCOOH	CO_2_ + 2H^+^ + 2e^−^ → HCOOH	−0.61	(6)
H_2_	2H^+^ + 2e^−^ → H_2_	−0.41	(7)

Porous materials exhibit a similar photocatalytic process to that of inorganic semiconductors. Taking MOFs as an example, compared with the inorganic semiconductors, the VB and CB of inorganic materials equal to the highest occupied molecular orbitals (HOMO) and the lowest unoccupied molecular orbitals (LUMO), respectively (Figure 2b). The following features make porous materials promising candidate for CO_2_ photoreduction:^[^ ^50^, ^51^ ^]^ i) High CO_2_ adsorption capacity makes the reaction site active to the adsorbed CO_2_ molecules, thus facilitates the performance of photocatalytic reduction of CO_2_. ii) The special porous structures have a pore confinement effect that will boost the catalysis process to a great degree. However, the performance of CO_2_ photoreduction still suffers low efficiency owing to three key aspects: insufficient utilization of visible‐light, negative electron–hole separation, and high inertness of active sites. Thus, in the next part, we summarize the existing strategies to improve the photocatalytic efficiency for CO_2_ reduction through tackling those three critical challenges.

## 3 Three Critical Aspects of MOFs‐Based Materials for CO_2_ Photoreduction

MOFs, a typical category of porous materials and built up with organic ligands and metal ions of clusters, have been extensively explored for CO_2_ photoreduction. Both the organic ligands and metal clusters can be a light harvest center owing to the metal complex like the inorganic semiconductor quantum dots (QDs), while the organic ligands can be considered antennae to harvest light.^[^ ^52^, ^53^ ^]^ Charge carrier separation and migration are vital for the reactions with the adsorbed molecules on the surface. Some sound strategies were introduced to ameliorate the charge carrier dynamics. Afterward, we discussed the correlation about the absorption of CO_2_ coupled with the activity of CO_2_ reduction.

### 3.1 Light Absorption

To expand the range of visible light absorption of MOFs materials, a number of strategies, such as amino‐modified, photosensitizer‐functionalized, electron‐rich conjugated linkers, post synthetic modifications (PSMs), and post synthesis exchange (PSE) were postulated. For metal clusters, metals were replaced with nonferrous ones or doping other ones to generate light harvest center.

#### 3.1.1 Amino‐Functionalized CO_2_ Reduction Photocatalysts

Since Garcia and co‐workers reported the use of MOF‐5 as a semiconductor to play charge‐separation under light irradiation,^[^ ^54^ ^]^ a number of publications regarding the semiconductor properties of MOFs have been explored.^[^ ^55^, ^56^, ^57^ ^]^ However, most MOFs exhibit poor conductivity due to the mismatch between the orbitals of leaker and metal, resulting in a short transfer distance. As above‐mentioned, organic leaker acts as antennae and transfers the generated electrons to metal clusters, namely linker‐to‐metal cluster charge transfer (LMCT), which provides a short‐range transfer of photogenerated electrons. Therefore, it is crucial for the leaker to produce photogenerated electrons and thus provide sufficient electrons to participate the photocatalysis process.

**Figure 3:**
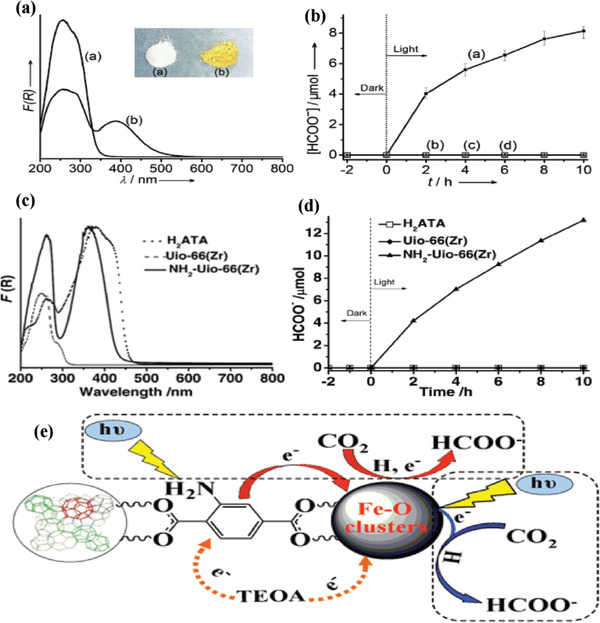
UV/vis spectra of a) MIL‐125(Ti) and b) NH_2_‐MIL‐125(Ti). The inset is the optical image of samples. b) Formate ion production rate of a) NH_2_‐MIL‐125(Ti) and b) MIL‐125. Reproduced with permission.^[^ ^58^ ^]^ Copyright 2012, Wiley‐VCH. c) UV/vis spectra of H_2_ATA, UiO‐66(Zr), and NH_2_‐UiO‐66(Zr). d) The formate ion production rate of samples. Reproduced with permission.^[^ ^59^ ^]^ Copyright 2013, Wiley‐VCH. e) Schematic illustration of Fe‐based MOFs for CO_2_ photoreduction. Reproduced with permission.^[^ ^60^ ^]^ Copyright 2014, Wiley‐VCH.

NH_2_‐functionalized leakers could greatly widen the range of optical absorption. Li and co‐workers reported a visible light responsive NH_2_‐MIL‐125(Ti) by a substitution of ligands NH_2_‐BDC for BDC leaker of MIL‐125(Ti),^[^ ^58^ ^]^ which broadens the absorption edge from 350 nm for MIL‐125(Ti) to 550 nm for NH_2_‐MIL‐125(Ti) (**Figure** **3**a). The formate evolution rate of NH_2_‐MIL‐125(Ti) and MIL‐125(Ti) was 16.28, ≈0 µmol h^−1^ g^−1^, respectively (Figure 3b). Later, the same group also explored the visible light responsive of NH_2_‐UiO‐66 by substituting NH_2_‐BDC linkers for BDC linkers.^[^ ^59^ ^]^ The absorption edge was increased to about 430 nm, which enhanced the photocatalytic activity (Figure 3c,d). It is noted that NH_2_‐UiO‐66(Zr) with mixed leakers even shows an improved formate generation rate. Obviously, (NH_2_)_2_‐BDC (DTA) partially replaces NH_2_‐BDC (ATA) in NH_2_‐UiO‐66(Zr) leading to enhancement in light absorption and CO_2_ absorption, which can improve the performance of photocatalytic reduction of CO_2_ with MOFs. Similarly, Wang et al. reported that all three NH_2_‐functionalized Fe‐based MOFs (NH_2_‐MIL‐101(Fe), NH_2_‐MIL‐53(Fe) and NH_2_‐MIL‐88B(Fe)) exhibited higher photocatalytic activity.^[^ ^60^ ^]^ However, these cases differ from above mentioned MIL‐125 or UiO‐66 with −NH_2_‐free modification. The NH_2_‐free Fe‐based MOFs exhibit semiconductor likewise in the absence of LMCT and are able to produce formate form under visible‐light irradiation. Nevertheless, after −NH_2_ modification, not only the light absorption edges of these Fe‐based MOFs were extended to nearly 700 nm, but their flat band potentials became more negative than that of bare MOFs according to the Mott–Schottky analysis. The possible mechanism is that –NH_2_ acts a photoexcited center, which facilitates photogenerated electrons to transfer to Fe center other than the direct photoexcitation of Fe–O clusters (Figure 3e).

Above‐mentioned studies demonstrate that NH_2_‐functionalized MOFs are a promising option to extend visible‐light range. However, most NH_2_‐functionalized MOFs exhibit light absorption limited to the range less than 550 nm. For instance, NH_2_‐MIL‐125(Ti)^[^ ^58^ ^]^ and NH_2_‐UiO‐66(Zr)^[^ ^59^ ^]^ expand the light absorption edge to nearly 550 and 450 nm, respectively. Therefore, some reports utilized conjugated molecules and amino groups together to further improve light responsive range of photocatalysts. By introducing functionalized conjugated ligand (H_2_L = 2,2′‐diamino‐4,4′‐stilbenedicarboxylic acid, H_2_SDCA‐NH_2_) into porous UiO‐type MOF, denoted as Zr‐SDCA‐NH_2_.^[^ ^61^ ^]^ Zr‐SDCA‐NH_2_ illustrates a broad‐band absorption edge at about 600 nm, exhibiting a formate evolution rate of 96.2 µmol h^−1^ mmol _MOF_^−1^. This study provides a new platform for effectively improving light absorption of MOFs through employing molecular conjugation.

#### 3.1.2 Electron‐Rich Conjugated Linkers CO_2_ Reduction Photocatalysts

**Figure 4:**
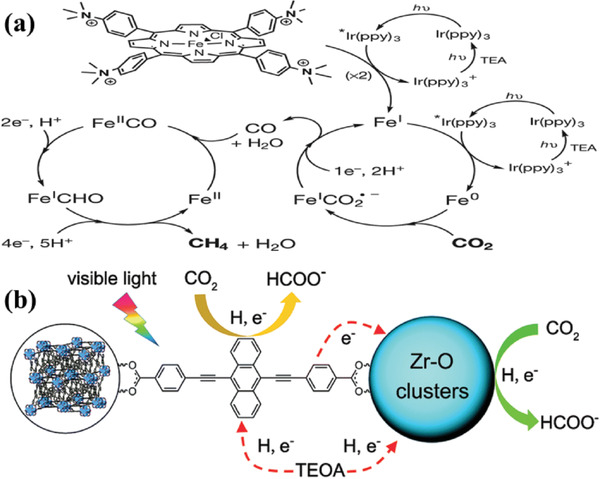
a) The mechanism for CO_2_ reduction to CH_4_ by catalyst. Reproduced with permission.^[^ ^64^ ^]^ Copyright 2017, Nature publishing group. b) The mechanism of NNU‐28 for visible‐light‐driven reduction. Reproduced with permission.^[^ ^67^ ^]^ Copyright 2016, Royal Society of Chemistry.

Electron‐rich conjugated linkers can improve the CO_2_ absorption capacity, and increase the light response range.^[^ ^62^, ^63^ ^]^ Porphyrin‐based ligand (H_2_TCPP) is constructed from four pyrrole rings, exhibiting a near‐planar 18 π‐conjugated network, which may be beneficial for the porphyrin‐based material for CO_2_ capture and conversion. Robert and co‐workers^[^ ^64^, ^65^ ^]^ reported an iron tetraphenylporphyrin complex modified with four trimethylammonio groups, exhibiting excellent performance for converting CO_2_ to CO or CH_4_ and the mechanism was also proposed as depicted in **Figure** **4**a. These investigations employed earth‐abundant Fe‐based materials with a cost‐effective nature. Similarly, Sadeghi et al. prepared a H_2_TCPP‐based MOF (Zn/PMOF) for the photocatalytic conversion of CO_2_ into CH_4_ in the presence of H_2_O vapor as a sacrificial agent.^[^ ^66^ ^]^ The CH_4_ production rate was 8.7 µmol h^−1^ g^−1^ and no by product was detected.

Anthracene‐based linker also exhibits excellent light absorption for electron‐rich conjugated structures. Su et al. reported that 4,4‐(anthracene‐9,10‐diylbis (ethyne‐2,1‐diyl)) dibenzoic acid reacts with ZrCl_4_ to form Zr‐MOF NNU‐28 ([Zr_6_O_4_(OH)_4_(L)_6_]·6DMF).^[^ ^67^ ^]^ NNU‐28 displays the highest formate production rate of 52.8 µmol g^−1^ h^−1^ in terms of Zr‐MOFs, which is attributed to the role of anthracene‐based ligand. In comparison with the organic ligand of H_2_ATA, anthracene‐based ligand serves as an antenna for light harvesting and participates in CO_2_ reduction reaction by radical formation (Figure 4b). However, H_2_ATA ligand shows no other extra contribution to the reaction, which provides a novel way to design visible‐light responsive MOFs‐based photocatalysts. Huang and co‐workers^[^ ^68^ ^]^ prepared porphyrin‐based Al‐PMOF coupled with Cu^2+^ and utilized it for photoreduction of CO_2_ into CH_3_OH, achieving high CH_3_OH formation rate of Al PMOF with Cu^2+^ (262.6 ppm g^−1^ h^−1^). This work provides a new strategy for designing efficient porphyrin‐based photocatalysts for capture and conversion of CO_2_ into liquid fuels.

#### 3.1.3 Photosensitizer‐Functionalized CO_2_ Reduction Photocatalysts

In addition to ligands, photosensitizer can also be modified to improve the photoresponse range of catalysts. The introduction of photosensitizers (Re^I^(CO)_3_(bpy)X complexes with bpy = 2,2′‐bipyridine and X = halide) into MOFs could harvest light and reaction centers. In general, 2,2′‐bipyridine‐5,5′‐dicarboxylic acid (5,5′‐dcbpy), 2,2′‐bipyridine‐4,4′‐dicarboxylic acid (4,4′‐dcbpy) and bpy units are ideal linkers for building photosensitizers given their promising coordination ability, which is analogue to a BPDC leaker with transition metal carbonyl complexes. Recently, a large number of research groups are exploring photosensitizer‐functionalized MOFs as photocatalysts for CO_2_ reduction, such as Ru–MOF (Y[Ir(ppy)_2_(4,4′‐dcbpy)]_2_[OH]), Ir‐CP ({Cd_2_[Ru(4,4′‐dcbpy)_3_]·12H_2_O}*_n_*).^[^ ^69^, ^70^ ^]^ In 2011, Lin and co‐workers reported visible‐light responsive UiO‐67 applied in CO_2_ reduction under light irradiation through incorporating a photosensitizer of Re^I^(CO)_3_(bpy)Cl.^[^ ^71^ ^]^

**Figure 5:**
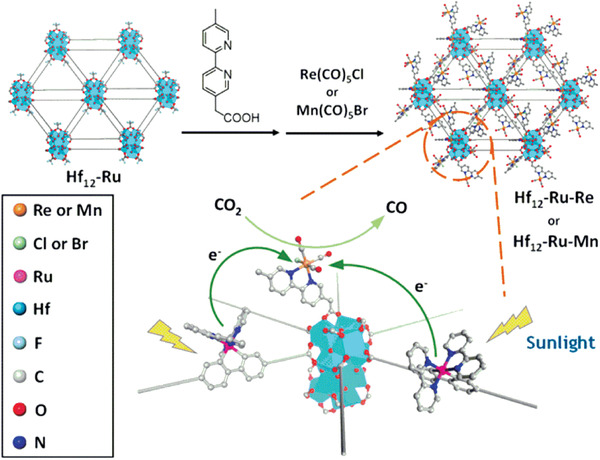
Schematic showing the synthesis of Ru‐Hf_12_‐M (M = Re or Mn) and the mechanism of photocatalytic CO_2_ reduction.^[^ ^72^ ^]^ Reproduced with permission.^[^ ^72^ ^]^ Copyright 2018, American Chemical Society.

However, CO evolution rate was relatively low even under the conditions of sacrificial agent of triethylamine (TEA). Later, they also reported photosensitizing metal–organic layers (MOLs) (Hf_12_‐Ru, based on Hf_12_ secondary building units (SBUs) and [Ru(bpy)_3_]^2+^ (bpy = 2,2′‐bipyridine) derived dicarboxylate ligands) as a new 2D material. Combining with photosensitizer M(bpy)(CO)_3_X (M = Re and X = Cl or M = Mn and X = Br), the complex exhibits efficient photocatalytic CO_2_ to CO^[^ ^72^ ^]^ (**Figure** **5**). These series of work demonstrate that incorporation of noble metal‐based photosensitizers into MOFs as building blocks is a sound approach for photocatalytic CO_2_ reduction.

#### 3.1.4 Postsynthesis of Exchange (PSE) or Metal Doping CO_2_ Reduction Photocatalysts

**Figure 6:**
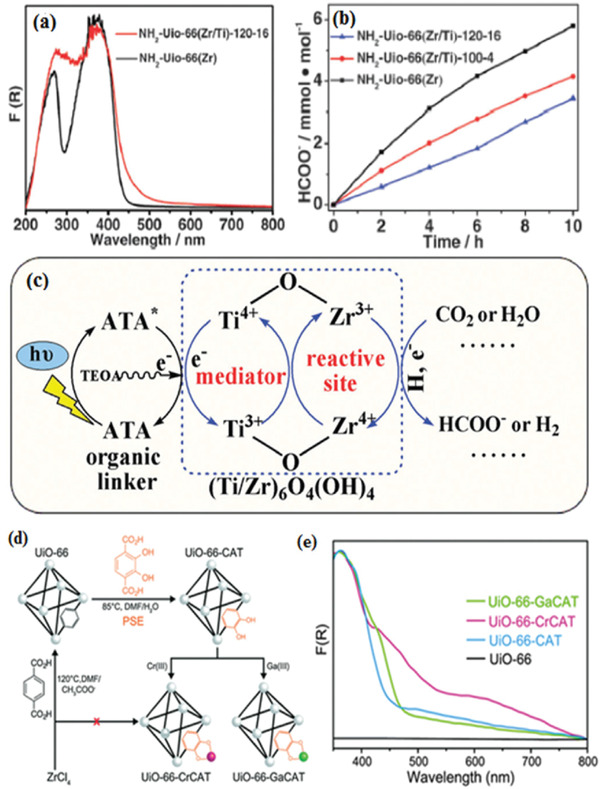
a) UV–vis spectra of the as‐prepared samples. b) The evolution rate of formate. c) Possible mechanism of Ti‐doping NH_2_‐UiO‐66(Zr/Ti). Reproduced with permission.^[^ ^75^ ^]^ Copyright 2015, Royal Society of Chemistry. d) Preparation of MOF photocatalysts by PSE. e) UV–vis spectra of as‐prepared samples. Reproduced with permission.^[^ ^76^ ^]^ Copyright 2015, Royal Society of Chemistry.

Apart from functionalized organic ligands, it is possible to enhance visible‐light responsiveness and photocatalytic performance of MOFs via functionalization of metal centers. As mentioned before, metal clusters resemble inorganic semiconductor quantum dots and organic ligands play as antennae to harvest light. The methods of PSMs and PSEs represent typical doping methods in semiconductor‐based photocatalysts, which may generate doped level or provide active photocatalytic sites to improve the overall efficiency of photocatalysis.^[^ ^73^, ^74^ ^]^ In 2015, Li and co‐workers was the first to synthesize Ti‐doping NH_2_‐UiO‐66(Zr/Ti) by using PSE, which enhanced photocatalytic activity for CO_2_ reduction and hydrogen production under visible light irradiation.^[^ ^75^ ^]^ As shown in **Figure** **6**a, the UV–vis spectra of Ti‐doping indicate an enhanced visible light absorption at the wavelength of 400 to 600 nm, leading to a high formate evolution rate (Figure 6b). The possible mechanism is illustrated in Figure 6c. ATA generates photoelectrons under light irradiation, which are then transferred to Ti–Zr–O oxo–metal clusters. This work is the first example to improve photocatalytic performance by means of PSE, which provides a generic method to explore excellent MOF‐based photocatalysts. However, HCOO^−^ evolution rate of Ti–Zr–O oxo–metal clusters needs further improvement compared pure NH_2_‐UiO‐66(Zr).

Pure UiO‐66(Zr) remains inactive for photocatalytic CO_2_ reduction up to date due to UV‐responsive and inefficient electrons transfer from leaker to metal clusters. In 2015, Cohen et al. ^[^ ^76^ ^]^ synthesized UiO‐66‐CAT (H_2_BDC replaced by 2,3‐dihydroxyterephthalic acid) and Cr‐monocatecholato species UiO‐66‐CrCAT, Cr‐monocatecholato species UiO‐66‐GaCAT through PSE (Figure 6d). The UV–vis spectra of the samples were shown in Figure 6e, UiO‐66‐CrACAT exhibited obvious visible light absorption because –OH increases HOMO level of H_2_BDC.^[^ ^77^, ^78^ ^]^ Photocatalytic performance reveals that UiO‐66‐CrACAT shows the highest formate evolution turnover number and presents a high stability. This work makes use of nonprecious metals (Cr) instead of noble metals (Ir, Pd) as dopants, providing a general way to develop more efficient catalysts.

### 3.2 Carrier Dynamics

The dynamics of charge carrier includes charge separation, migration, mobility, and diffusion length, which is one of the critical aspects in determining the efficiency of photocatalysis. In principle, photocatalytic reaction occurs only when photogenerated electrons are transferred to the surface. However, in many photocatalysts including porous materials or nonporous materials, the photogenerated electrons are recombination with holes. Only a small number of photoelectrons can participate in the process of photocatalysis. Therefore, it is extremely important to investigate the charge carrier dynamics. In this section, MOFs are coupled with other cocatalysts or semiconductors to form heterojunction or electron trapping sites, leading to efficient charge separation.

#### 3.2.1 MOFs Coupled with Semiconductors as Photocatalysts

The acceleration of charge carrier separation and inhibition of harmful charge recombination is the key to improve the photocatalytic efficiency for photocatalytic CO_2_ reduction.^[^ ^79^ ^]^ MOFs coupled with other materials may form heterojunction or electron capture sites in composite materials, which enable photogenerate electrons transfer from one part to another, leading to effective charge separation and improved catalytic activity.

In 2013, Liu et al. ^[^ ^80^ ^]^ first synthesized the composites of Zn_2_GeO_4_ and ZIF‐8 (zinc containing ZIFs) for efficient photocatalytic conversion of CO_2_ into liquid CH_3_OH, which is attributed to the efficiency carrier separation by forming heterojunction. Such a promising strategy is a key for investigating highly efficient photocatalysts to improve CO_2_ reduction efficiency by the virtue of excellent adsorption property of MOFs in aqueous media. After that, Wang and co‐workers systematically studied ZIF‐9 coupled with semiconductors such as CdS,^[^ ^81^ ^]^ C_3_N_4_^[^ ^82^ ^]^ to ameliorate the charge transfer in the CO_2_ photoreduction process, which greatly improved the overall photocatalytic performance. Ye and co‐workers synthesized Co‐ZIF‐9/TiO_2_ nanocomposites for photocatalytic CO_2_ reduction.^[^ ^83^ ^]^ The results of transmission electron microscopy (TEM), and X‐ray photoelectron spectroscopy (XPS) of the optimal sample show TiO_2_ and ZIF‐9 are very close with each other, leading to efficient separation of carrier. The highest photocurrent density of optimal further confirms the best carrier efficiency. This work presents that fabrication of Co‐ZIF‐9 with semiconductors to form a well‐designed structure is vital to further improve the performance of CO_2_ photoreduction.

**Figure 7:**
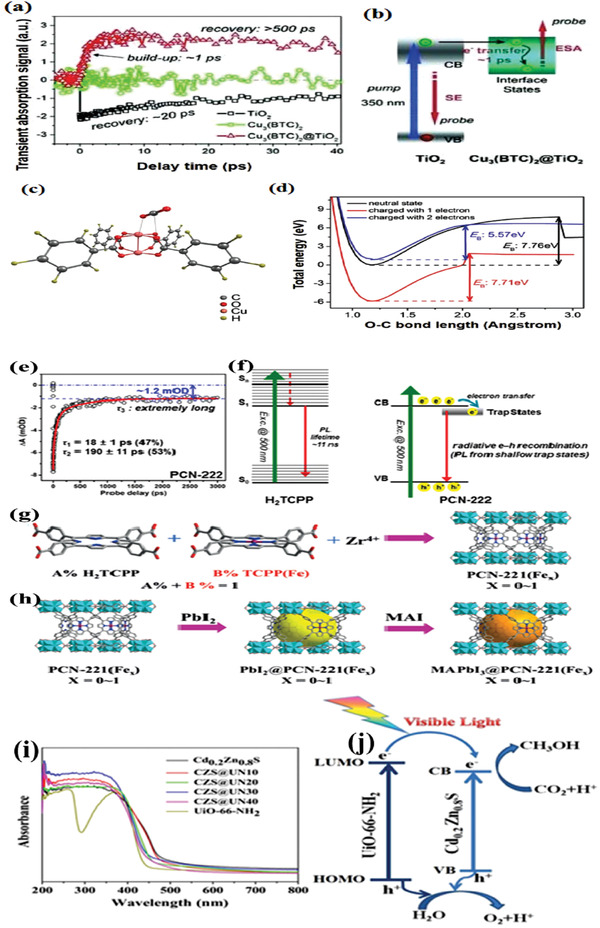
a) Ultrafast transient absorption and b) photoexcited dynamics. c) The optimized structure of a CO_2_ molecule adsorbed on Cu_3_(BTC)_2_. d) Change of band energy *E*_B_ for CO_2_ reduction after the addition of one or two‐electron charge. Reproduced with permission.^[^ ^84^ ^]^ Copyright 2014, Wiley‐VCH. e–f) TA spectra and carrier dynamics of H2TCPP, PCN‐222. Reproduced with permission.^[^ ^86^ ^]^ Copyright 2015, American Chemical Society. g,h) Schematic illustrations of encapsulated MAPbI3@PCN‐221(Fex). Reproduced with permission.^[^ ^87^ ^]^ Copyright 2019, Wiley‐VCH. i) UV–vis spectra of pure UiO‐66‐NH_2_, Cd_0.2_Zn_0.8_S, and CZS@UN composites. j) Schematic depict of charge carrier separation of composites for CO_2_ photoreduction. Reproduced with permission.^[^ ^90^ ^]^ Copyright 2017, Elsevier.

Similarly, Li et al. prepared core–shell‐structured Cu_3_(BTC)_2_@TiO_2_ (BTC = 1,3,5‐benzenetricarboxylate) for photocatalyst in CO_2_ reduction.^[^ ^84^ ^]^ It was designed for photocatalytic reduction of CO_2_ to CH_4_ in the presence of water, which also acts as sacrificial donor. In the composite photocatalyst, a large surface area of MOF plays as core and provides adsorption and photoconversion to CO_2_ molecules. Shell of the macroporous TiO_2_ is a semiconductor for supplying photogenerated electrons, which is easy to be excited by light and easy to diffuse in a MOF based core. In order to examine the carrier dynamics of the samples, ultrafast transient absorption (TAS) was carried out. As shown in **Figure** **7**a,b, the electrons were transferred from TiO_2_ to the electrons trapping sites (Cu_3_(BTC)_2_) efficiently, leading to improvement in carrier separation.

Density functional theory (DFT) calculations presented that two photogenerated electrons transferred from TiO_2_ to Cu_3_(BTC)_2,_ leading to high adsorption energy of CO_2_ and reducing *E*_B_ from 7.76 eV for neutral state to 5.57 eV for charged with two electrons (Figure 7c,d), which facilitated the CO_2_ adsorption by Cu_3_(BTC)_2_. Similarly, Crakea et al. prepared TiO_2_/NH_2_‐UiO‐66 heterostructures via an in situ process.^[^ ^85^ ^]^ The close contact between TiO_2_ and NH_2_‐UiO‐66 facilitated electron migration, and rendered high efficiency of electron hole separation. The efficient charge transfer was further confirmed by TAS spectroscopy.

Porphyrin‐based semiconducting MOF PCN‐222 was used in CO_2_ photoreduction for the first time in 2015,^[^ ^86^ ^]^ exhibiting higher activity than that of H_2_TCPP leaker alone. As shown in Figure 7e,f, TA and photoluminescence spectra demonstrate that PCN‐222 has a long‐lived electron trap state, thus inhibits the electron–hole recombination and yields high efficiency of CO_2_ photoreduction. PCN‐222 exhibited much better activity than that of H2TCCP leaker alone. This work not only provides a new understanding of the carrier dynamics involved in MOFs, but also unveils the mechanism of charge‐carrier transfer. Recently, Zhang et al. reported a MAPbI_3_@PCN‐221(Fe*_x_*) composite for CO_2_ photoreduction. As illustrated in Figure 7g,h, MAPbI_3_ was encapsulated in the pores of PCN‐221(Fe*_x_*),^[^ ^87^ ^]^ which is beneficial to the effective transfer of photogenerated electrons from the encapsulated MAPbI_3_ QDs to Fe catalytic sites, leading to high charge separation efficiency. This current study provides a method to improve the stability of lead halide perovskite QDs in aqueous atmosphere.

Transition metal sulfides (TMSs) were widely studied for photoreduction of CO_2_ in recent years owing to visible‐light responsiveness, low cost and satisfactory performance.^[^ ^88^, ^89^ ^]^ A series of nanocomposites were prepared by incorporating different contents of UiO‐66‐NH_2_ with solid‐solution Cd_0.2_Zn_0.8_S for photocatalytic CO_2_ reduction.^[^ ^90^ ^]^ UV–vis spectrum reveals that the absorption edge of composites achieved slight red shift (Figure 7i), suggesting that composites are more responsive to sunlight. Of the as‐synthesized samples, an optimal composite (CZS@UN20, 20 wt% of UiO‐66‐NH_2_) exhibits the highest CH_3_OH evolution rate of 6.8 µmol h^−1^ g^−1^ under visible‐light irradiation, in which the CH_3_OH production rate of pure Cd_0.2_Zn_0.8_S is only 2.0 µmol h^−1^ g^−1^. The remarkable enhancement in performance was mainly attributed to efficient charge carrier separation (Figure 7j). With visible‐light irradiation, photogenerated electrons transferred from UiO‐66‐NH_2_ to Cd_0.2_Zn_0.8_S since the LUMO potential of UiO‐66‐NH_2_ was more negative than that of Cd_0.2_Zn_0.8_S, inhibiting the electron–hole recombination, thus achieved high activity of CO_2_ photoreduction. This work offers a promising candidate to practical applications. Therefore, the heterojunction composite materials formed by MOF and semiconductor materials can effectively separate photogenerated carriers. However, due to the inherent defects on the surface of inorganic semiconductor cannot be well grafted tightly with MOFs, many literatures used this method to greatly improve the efficiency of photogenerated charge separation which needed to be verified.

Using likewise doping method to synthesize the so‐called “multi‐metal‐site” catalysts, it is feasible to form doping energy levels in the metal cluster center, like inorganic semiconductors, which is not only conducive to light absorption, but also can provide the active site or acted as capture carrier center, so as to improve the overall efficiency of photocatalytic reduction of CO_2_. However, up to now, there is no in‐depth study at atomic level of polymetallic MOF to find out how the doping could generate defects in the structure, which is a hot spot in recent years but a difficult problem.

#### 3.2.2 MOFs Coupled with Metal as Photocatalysts

**Figure 8:**
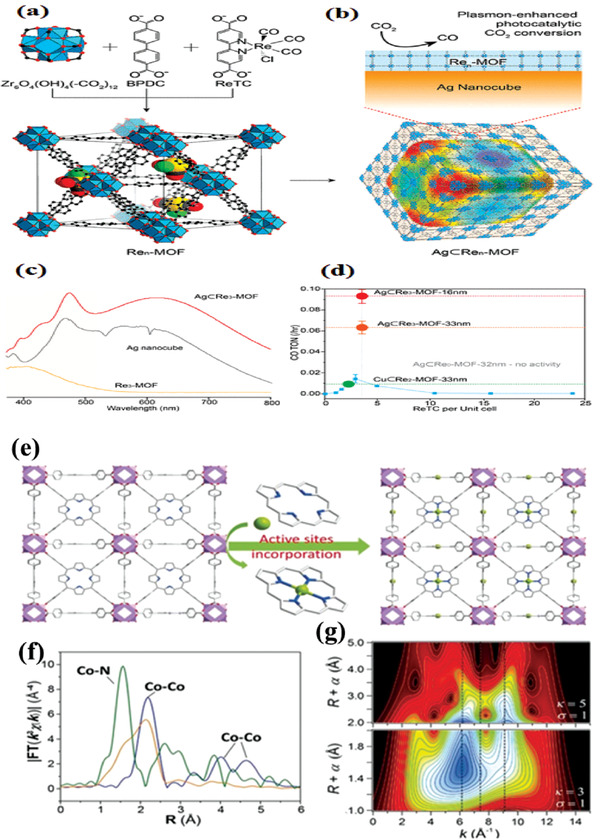
a) Zr_6_O_4_(OH)_4_(−CO_2_)_12_ secondary building units and the formation schematic of Re*_n_*‐MOF. b) Re*_n_*‐MOF coated on Ag nanocube. c) UV–vis spectra of Re_3_‐MOF, Ag nanocube, and Ag⊂Re_3_‐MOF. d) Photocatalytic CO_2_‐to‐CO conversion activity of Re*_n_*‐MOFs (blue line), Ag⊂Re_0_‐MOF, Cu⊂Re_2_‐MOF, and Ag⊂Re_3_‐MOFs with MOF thickness of 16 and 33 nm.^[^ ^92^ ^]^ Reproduced with permission.^[^ ^92^ ^]^ Copyright 2017, American Chemical Society. e) Schematic depicts of preparation of MOF‐525‐Co and f) Fourier transform magnitudes of the experimental Co K‐edge EXAFS spectra of samples. g) Wavelet transform for the k^3^‐weighted EXAFS signal of MOF‐525‐Co.^[^ ^99^ ^]^ Reproduced with permission.^[^ ^99^ ^]^ Copyright 2016, Wiley‐VCH.

Metal is utilized widely in photocatalysis as a light harvest center owing to their surface plasmon resonance (SPR) effect. Of those, double‐shelled plasmonic Ag–TiO_2_ hollow spheres are a good example for improving the activity of CO_2_ photoreduction.^[^ ^91^ ^]^ Similarly, MOFs are a category of materials with porous structures that are elegant host matrices in confining versatile functional guest species, such as metal nanoparticles (MNPs), to yield enhanced photocatalytic performance through a synergistic effect.^[^ ^92^ ^]^ A latest report by Yaghi and co‐workers presented such an attempt to the employment of MNPs/MOFs (Ag‐nanocubes‐MOF core–shell composites, Ag⊂Re*_n_*‐MOF) for photocatalytic reduction of CO_2_.^[^ ^92^ ^]^ Re(CO)_3_(bpydc)Cl, a catalytic center, was attached to linkers to produce Re‐UiO‐67, followed by coating upon Ag nanocubes to yield Ag⊂Re‐UiO‐67 (**Figure** **8**a,b). It is apparent that thickness of the Re complexes contributes greatly to the catalytic activity. As presented in Figure 8c,d, Re complexes with a thickness of 16 nm, i.e., Re_3_‐MOF‐16 nm, plays the highest photocatalytic activity. Moreover, Ag⊂Re_3_‐MOF‐16 nm retains the original SPR features, thus produces a strong electromagnetic filed to confine spatially photoactive Re metal sites in the shell, and consequently exhibits seven times higher enhancement in photocatalytic evolution activity of CO_2_‐to‐CO than that of Re‐UiO‐67 under visible‐light irradiation. This study shows that covalently attached active centers within interior MOFs can be spatially localized and increase their photocatalytic performance by electromagnetic field induced by plasmonic silver nanocubes.

In a following study, ultrafine Ag NPs were doped into Co‐ZIF‐9 (Ag@Co‐ZIF‐9) for photocatalytic reduction of CO_2_ to CO under visible light in the presence of a photosensitizer.^[^ ^93^ ^]^ The photocatalytic performance of composites was enhanced twofold from that of Co‐ZIF‐9, demonstrating doping with MNPs was an efficient way to enhance photocatalytic efficiency of MOFs.

In addition, it is well recognized that the separation efficiency of charge carriers is an important factor to photocatalytic activity of semiconductor photocatalysts. When a Schottky barrier was formed at the junction of semiconductor and noble metal, photogenerated electrons in the semiconductor of a CB can be transferred to adjacent noble metal center, thus improving the separation of photogenerated carriers and ultimately improving the photocatalytic performance.^[^ ^94^, ^95^ ^]^ Therefore, doping precious metal, such as Pt and Au, into a semiconductor photocatalyst is a common method to suppress the recombination of photogenerated electrons and holes. Li and co‐workers^[^ ^96^ ^]^ studied the effects of different metal‐doped M‐NH_2‐_MIL‐125(Ti) (M = Pt and Au) photocatalysts on CO_2_ reduction. Compared with pure NH_2_‐MIL‐125(Ti), Pt‐doped exhibited a higher formate evolution rate while Au‐doped exhibited lower formate production, indicating noble metal could influence the electron‐trapping, which further changed the products. Interestingly, Au‐NH_2_‐MIL‐125 imposed a negative effect on photocatalytic formate production. To elucidate the mechanism of different photocatalytic activities, ESR and DFT calculations for M‐NH_2_‐MIL‐125 were carried out. Results indicate hydrogen could spill over from Pt to Ti atoms, leading to the formation of Ti^3+^, which was considered as the active sites to produce formate. However, hydrogen spillover was difficult over Au‐NH_2_‐MIL‐125(Ti). Therefore, a negative effect on the photocatalytic formate generation was detected over Au‐NH_2_‐MIL‐125(Ti), indicating that the selection of an appropriate noble metal is key to the desired photocatalytic activity. This work illustrates that an effective Schottky barrier can be formed only if their Fermi band potential between noble metals and semiconductors are considered.

In addition to noble metal, nonprecious metal can also be incorporated with MOFs to photocatalytically reduce CO_2_ in an efficient manner. In recent years, atomically dispersed catalysts such as so‐called “single atoms anchored on matrix” which utilized maximum atom efficiency.^[^ ^97^ ^]^ However, it is still challenging to fabricate practical and stable single atom catalysts owing to their high mobility in a catalytic process.^[^ ^98^ ^]^ In this case, porous materials are a good candidate as matrix for providing coordination sites to anchor single metal atoms. Ye and co‐workers synthesized atomic Co dispersion of active sites in MOF‐525.^[^ ^99^ ^]^ Co sites were incorporated into the porphyrin units to form MOF‐525‐Co and atomic Co was demonstrated by the Co K‐edge extended X‐ray absorption fine structure (EXAFS) and X‐ray absorption near‐edge structure (XANES) spectroscopy (Figure 8e–g). According to the energy transfer investigation coupled with the first principles calculation, photogenerated electrons could be effectively shifted to the reaction center Co “trap site,” which ameliorates charge separation, achieving CO evolution rate of 200.6 µmol g^−1^ h^−1^ and CH_4_ production rate of 36.67 µmol g^−1^ h^−1^, 3.13‐fold and 5.93‐fold from that of pure MOF. This work provides a strategy that could take advantages of the coordination feature of porous materials to design MOF‐based photocatalysts through efficient atomic doping for CO_2_ reduction.

### 3.3 Adsorption/Activation and Reaction with CO_2_

**Scheme 2:**
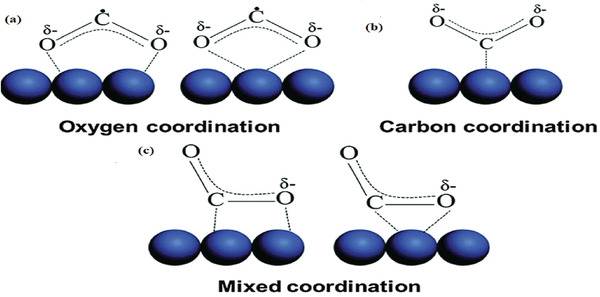
Three possible coordination ways of CO_2_ on the catalyst surface. a) Oxygen coordination, b) carbon coordination, and c) mixed coordination. Reproduced with permission.^[^ ^100^ ^]^ Copyright 2016, Royal Society of Chemistry.

In general, a catalytic reaction requires the substrates adequately adsorbed on the surface of catalysts. Regarding photocatalytic CO_2_ reduction, adsorption of CO_2_ molecules on the surface of catalysts is a prerequisite owing to the low solubility of CO_2_ in most liquid solutions. As shown in **Scheme** **2**, three possible coordination structures of adsorbed CO_2_ on the surface of a catalyst were proposed. Firstly, oxygen of CO_2_ has a long pair of electrons that can coordinate with Lewis acid centers on the surface (Scheme 2a). Similarly, carbon in CO_2_ acted as Lewis acid that could donated to Lewis base centers on the surface (Scheme 2b). In the third type, oxygen and carbon are mixed coordinated with surface Lewis acid and Lewis base centers, respectively (Scheme 2c).^[^ ^100^ ^]^ On one hand, MOF has multifunctional ligands that can be modified to form basic sites to activate inert CO_2_ molecules. On the other hand, MOFs possess high porosity, large surface area and tunable structure such as replaced acidic leaker by basic leaker to adsorb more CO_2_ molecules, thus facilitate photocatalysis. However, the concentration of CO_2_ in atmosphere is quite low and to capture CO_2_ from air will cost a lot of energy. Therefore, understanding how CO_2_ uptake capacity of porous materials affects their photocatalytic CO_2_ reduction is important for developing more efficient photocatalysts under low concentration of CO_2_. It will be practical in industrial if flue gas can be directly used as CO_2_ feedstocks.

**Figure 9:**
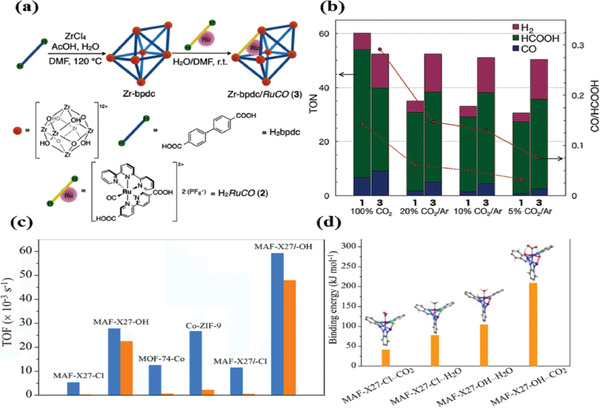
a) Synthesis of UiO‐67/RuCO, UiO‐67. b) The relationship between photocatalytic activity and CO_2_ pressure. Reproduced with permission.^[^ ^101^ ^]^ Copyright 2016, Wiley‐VCH. c) TOF value under 0.1 atm of CO_2_. d) The binding structures and energies of MAF‐X27‐Cl and MAF‐X27‐OH. Reproduced with permission.^[^ ^102^ ^]^ Copyright 2018, American Chemical Society.

To understand the adsorption capacity of CO_2_ and activation of inert CO_2_ molecules, it is essential to unveil the relationships between the uptake capacity of CO_2_ and performance. For instance, Ru^II^‐CO complex ([Ru^II^(bpy)(terpy)(CO)](PF_6_)_2_) was synthesized using a PSE method with UiO‐67^[^ ^101^ ^]^ (**Figure** **9**a). As shown in Figure 9b, the photocatalytic activity decreases with decreasing partial pressure of CO_2_, indicating that the photocatalytic activity was highly dependent on the concentration of CO_2_. In contrast, catalytic performance of UiO‐67/RuCO is close to that measured under 5% CO_2_ atmosphere, which demonstrates that the composite could effectively adsorb CO_2_ in dilute concentrations and use of synergy between the adsorptive sites and the catalytic active sites.

Recently, three isostructural MOFs including MAF‐X27‐Cl, MAF‐X27‐OH, MOF‐74‐Co were used for CO_2_ photoreduction.^[^ ^102^ ^]^ When the partial pressure was decreased to 0.1 atm, the photocatalytic activity of MAF‐X27‐Cl, MOF‐74‐Co decrease severely, while the MAF‐X27‐OH also exhibited a high CO TOF of 23 × 10^−3^ s^−1^ (28 × 10^−3^ s^−1^ at 1 atm). DFT simulations demonstrated that the m‐OH of MAF‐X27‐OH was coordinated with the open Co sites, which stabilized the Co–CO_2_ by hydrogen bonding, thus boosting the photocatalytic CO_2_ reduction (Figure 9c,d). Very recently, Wang and co‐workers synthesized UiO‐66/TiO_2_ composites^[^ ^103^ ^]^ which displayed high yields of CH_4_ even at diluted CO_2_ condition (≤2%), though the detailed mechanism was not clear yet.

**Figure 10:**
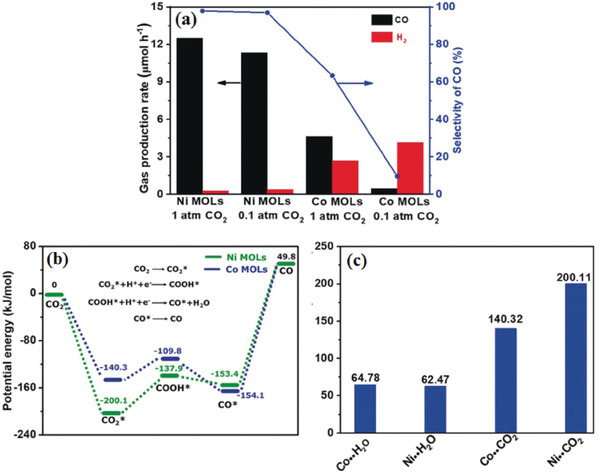
a) CO_2_ photoreduction activity of Ni MOLs and Co MOLs in pure CO_2_ and diluted CO_2_ (10 %). b) DFT calculation of active energy barrier of Ni MOLs and Co MOLs, respectively. c) CO_2_ and H_2_O adsorption energies of Ni MOLs and Co MOLs.^[^ ^39^ ^]^ Reproduced with permission.^[^ ^39^ ^]^ Copyright 2018, Wiley‐VCH.

Although many works tried to convert CO_2_ at low concentrations, this filed is still at its early stage, suffering low durability and low selectivity of CO_2_ reduction. Considering that, Lin and co‐workers^[^ ^39^ ^]^ synthesized a monolayer Ni MOFs, namely Ni MOLs, for photoreduction in diluted CO_2_, exhibiting CO generation rate of 12.5 µmol h^−1^, with a high CO selectivity of 97.8 %, much higher than that of Co MOLs (**Figure** **10**a). To elucidate such a phenomenon, DFT calculations were carried out. As presented in Figure 10b, the active energy barrier COOH* formation over Co MOLs was slightly smaller than that of Ni MOLs, indicating that Co MOLs were more favorable to COOH* formation than Ni MOLs in kinetics, which was contrary to the performance results. As shown in Figure 10c, the adsorption energy of CO_2_ on Ni MOLs is −200.11 kJ mol^−1^, which was much stronger than that of the Co MOLs (−140.32 kJ mol^−1^). Therefore, DFT results clearly demonstrated that the initial adsorption of CO_2_ on MOLs was the crucial step of the reaction system. It was also validated that the selectivity of photocatalytic reduction of CO_2_ is directly correlated with the binding affinity of CO_2_ molecules.

Abovementioned relationships between CO_2_ uptake capacity of MOFs photocatalyst and performance of CO_2_ photoreduction are summarized as follows: Firstly, the activity of photocatalytic reaction is positively related to the concentration of CO_2_. Secondly, some functional groups or a high diversity of metals in MOFs may activate CO_2_ molecules and improve CO_2_ uptake such as hydrogen bonding, thus improve photocatalytic activity. Thirdly, MOF‐based materials combining with high CO_2_ adsorption energies of catalysts enable high catalytic activity even in a diluted CO_2_ atmosphere.

## 4 Recent Advances of Other Porous Materials for Photocatalytic CO_2_ Reduction

In addition to MOF, other porous materials such as COFs‐based, zeolite‐based and inorganic/organic porous semiconductors are also used for photocatalytic reactions.

### 4.1 COF‐Based Photocatalysts for CO_2_ Reduction

Yaghi and co‐workers first reported COF‐1 via self‐condensing with phenyl diboronic acid.^[^ ^104^ ^]^ As a new class of porous material, COFs provide a versatile platform for CO_2_ photoreduction.^[^ ^105^, ^106^, ^107^, ^108^ ^]^ COFs are formed by periodic organic building blocks through covalent bonds. The establishment of spiropyrans (Ps) (i.e., extended π‐conjugation) promotes effective separation of charge carrier.

COFs have a well adjustable structure. Organic compounds are combined into the original counterpart to form a multifunctional COF photocatalyst with a dual function of redox and oxide. This kind of close connection way makes photogenerated charge transfer rapidly and reduces instability of photocatalysis to a certain extent. In recent years, there are many ligands modified to achieve effective separation of photogenerated charges.^[^ ^109^, ^110^ ^]^ Wisser et al.^[^ ^111^ ^]^ reported using chromophores as light harvest antenna (controlling HOMO) and Cp*Rh as catalytic sites (regulating LUMO), which realize the rapid transfer of photogenerated charge. This kind of unique structure of long‐term stable perylene photosensitizer and the selective Rh‐based catalyst Cp*Rh@PerBpyCMP made it possible for photoreduction of CO_2_ in several days. The yield of formate was about 65 mmol g_cat_^−1^, which is the highest value obtained in heterogeneous photocatalysis. Wang and co‐workers^[^ ^112^ ^]^ also reported a covalent triazine based framework (CTF) consisting of triphenylamine and triazine, which can be effectively used for photocatalytic reduction of CO_2_. The p‐conjugated structure provides a channel for the migration and separation of photoexcited electrons, which improves the photocatalytic activity. The self‐functionalized DA‐CTFs method not only improves the photocatalytic activity of organic semiconductors, but also cast new sights upon the fabrication of photocatalysts.

Su and co‐workers reported a pure TAPBB‐COF (synthesized with TAPP [5,10,15,20‐tetrakis(4‐aminophenyl)‐porphyrin] and 2,5‐dibromo‐1,4‐benzenedialdehyde) for CO_2_ photoreduction in presence of water and without any additional co‐reactants. By tuning the valence band of TAPBB‐COF, the photocatalyst achieved a high CO evolution rate of 295.2 µmol g^−1^.^[^ ^113^ ^]^ This was the first work for photocatalytic CO_2_ reduction using COFs along without any sacrificial donor or co‐catalysts.

**Figure 11:**
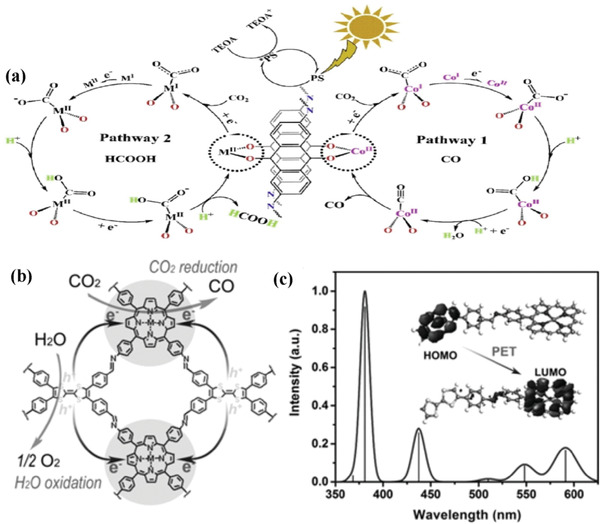
a) The photocatalytic schematic of DQTP‐COF‐M (M = Co, Zn). Reproduced with permission.^[^ ^114^ ^]^ Copyright 2019, Elsevier. b) The mechanism of TTCOF‐M CO_2_RR with H_2_O oxidation. c) DFT simulation UV/vis DRS of TTCOF‐Zn and scheme of PET route under light excitation (inset). Reproduced with permission.^[^ ^115^ ^]^ Copyright 2019, Wiley‐VCH.

In comparison with pure COFs, metalized‐COFs forming hybrid metal–complex systems often exhibited higher performance. Lan and co‐workers synthesized the first metalized‐COFs photocatalyst for CO_2_ photoreduction.^[^ ^114^ ^]^ As illustrated in **Figure** **11**a, the as‐synthesized DQTP‐COF‐Co(2,6‐diaminoanthraquinone (DQ), (TP) 2,4,6‐triformylphloroglucinol) exhibited excellent CO_2_ reduction activity coupled with photosensitizer Ru(bpy)_3_Cl_2_ and triethanolamine (TEOA) providing electron and protons, resulting in a high CO formation rate of 1020 µmol g^−1^ h^−1^.

Artificial photosynthesis is expected to use only H_2_O as electron sources in the absence of any additional sacrificial donor. To this end, Lan et al. developed a series of Z‐scheme porphyrin–tetrathiafulvalene COFs (TTCOF‐M, M = 2H, Zn, Ni, Cu) for photocatalytic CO_2_ reduction.^[^ ^115^ ^]^ In that composite, electron‐deficient TAPP has good visible‐light harvesting ability.^[^ ^116^ ^]^ Meanwhile, electron‐rich tetrathiafulvalene (TTF) has demonstrated to be an excellent electron donor.^[^ ^117^ ^]^ Therefore, it is possible to combine TAPP and TTF to form a Z‐scheme (TAPP and TTF act on the reduction site and oxidation site, respectively) to transfer photogenerated electrons from TTF to TAPP under visible light irradiation, and effectively separate electron holes (Figure 11b,c). As expected, TTCOF‐Zn exhibited the highest CO evolution rate of 12.33 µmol after 60 h with nearly 100% selectivity and good stability. This is the first report of COF composites applied in the overall reaction of CO_2_ with H_2_O without any extra photosensitizer or sacrificial donor. However, the CO production rate surfers very slow. The possible reasons might be that the oxidation ability of TTF is relatively weak. Therefore, incorporated with much stronger oxidation ability material may be a good strategy for further improving photocatalytic performance.

Although many COFs were reported for CO_2_ photoreduction, it is still worth to concern that a large enough 2D COF single‐crystal is extremely difficult to be obtained. As a result, the actual structure of COFs is determined only by powder XRD and computational simulation, which limits the effective structure–activity relationship analysis of COFS.

### 4.2 Zeolite‐Based Photocatalysts for CO_2_ Reduction

**Figure 12:**
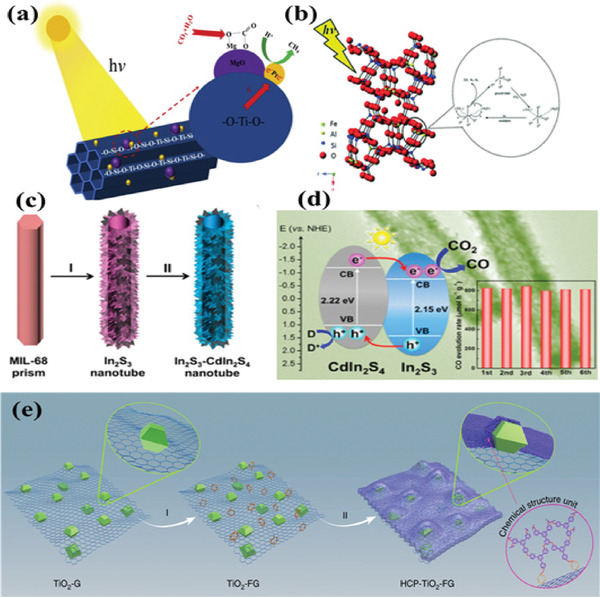
a) The proposed mechanism of CO_2_ reduction of Ti‐MCM‐41. Reproduced with permission.^[^ ^125^ ^]^ Copyright 2019, Elsevier. b) The possible mechanism of CO_2_ reduction of HZSM‐5. Reproduced with permission.^[^ ^126^ ^]^ Copyright 2016, Royal Society of Chemistry. c) Schematic illustration of the synthesis of In_2_S_3_‐CdIn_2_S_4_ heterostructured nanocube and d) its band structure and recyclability for photocatalytic CO_2_ reduction. Reproduced with permission.^[^ ^147^ ^]^ Copyright 2017, American Chemical Society. e) Construction of a well‐defined porous HCP‐TiO_2_‐FG composite structure. Reproduced with permission.^[^ ^149^ ^]^ Copyright 2019, Nature Publishing Group.

Compared with traditional semiconductors, molecular sieves are intrinsic porous structure with high specific surface area and many active sites for photocatalysis.^[^ ^118^, ^119^ ^]^ Anpo et al. took the lead in investigating a series of Titanium oxides anchored within zeolites. The highly dispersed TiO_2_ within Y‐zeolite cavities (Ti‐oxide/zeolite) were synthesized by an ion‐exchange method, achieving high selectivity to produce CH_4_. The charge excited of the important intermediates (Ti^3+^‐O^−^)* are generated under UV irradiation. The generated electrons were trapped into H^+^ and CO_2_ to form H atoms and CO, then produced a series of carbon radicals. Finally, the reaction of these radicals produced CH_3_OH and CH_4_.^[^ ^120^ ^]^ Next year, Ti‐MCM‐41 and Ti‐MCM‐48 were investigated in CO_2_ photoreduction.^[^ ^121^ ^]^ Bai and co‐workers^[^ ^122^ ^]^ first investigated using Ti‐MCM‐41 photocatalysts in monoethanolamine (MEA) solution for methane production. The optimal photocatalyst of Ti‐MCM‐41(50) (50 denote Si/Ti molar ratio of 50) exhibited CH_4_ yield of 62.42 µmol g^−1^ of cat after 8 h of UV irradiation. However, monoethanolamine was used as sacrificial agent, which was not environmentally and energy saving. Other than parent molecule severs, the zeolite‐based composites were also investigated in recent years. Cu–porphyrin impregnated mesoporous Ti‐MCM‐48 was investigated for the CO_2_ reduction under visible light irradiation, exhibiting methanol yield of 85.88 µmol g^−1^ L^−1^.^[^ ^123^ ^]^ Yang and co‐workers investigated the Pt/MgO loaded Ti‐MCM‐41 zeolite with different Si/Ti molar ratios for photocatalytic CO_2_ reduction.^[^ ^124^ ^]^ The electrons and holes were photogenerated in TiO_4_ tetrahedral units in molecular sieve under light irradiation, leading to form [Ti^3+^‐O^−^]^∗^ (**Figure** **12**a). The high photocatalytic activity was achieved on Ti‐MCM‐41 because of synergistic effect. HZSM‐5 zeolites were used in CO_2_ photoreduction for the first time by Wang and co‐workers^[^ ^125^ ^]^ [Fe^3+^–O^2−^] species could be excited by UV light to form an important intermediate, [Fe^2+^–O^−^]*, achieving high photocatalytic activity (Figure 12b). Very recently, Jing and co‐workers reported that composites of optimal Ag‐modified 2D/2D hydroxylated g‐C_3_N_4_/TS‐1 exhibited sevenfold than that of 2D TS‐1.^[^ ^126^ ^]^ The enhanced photoactivity is attributed to the Z‐scheme mechanism between hCN and TS‐1, which greatly enhanced charge separation and extended range of visible‐light absorption. This work presented a feasible design strategy to synthesize high efficiency TS‐1 zeolite‐based photocatalyst.

Although many zeolites have been used in photocatalytic CO_2_ reduction, there are still many problems to be solved. For example, structure–activity relationship is still unclear. The overall yield is still very low. Much more novel molecular sieves need to be developed for photoreduction of CO_2_.

### 4.3 Inorganic/Organic Porous Semiconductors

In addition to the classic porous materials for the CO_2_ photoreduction, inorganic/organic porous materials were also discussed in this section.^[^ ^127^, ^128^, ^129^, ^130^, ^131^, ^132^ ^]^ For the CO_2_ reduction, an efficient photocatalyst should possess high uptake capacity of CO_2_. Therefore, porous carbon materials, porous metal oxides, are discussed in this section. Wang et al. ^[^ ^133^ ^]^ synthesized hybrid carbon@TiO_2_ hollow sphere by utilizing a template of a carbon nanosphere. The optimal composites exhibited CH_4_ evolution rate of 4.2 µmol g^−1^ h^−1^ and CH_3_OH production rate of 4.2 µmol g^−1^ h^−1^. The enhanced photoactivity was attributed to the increased CO_2_ uptake (0.64 mmol g^−1^) and specific surface area (110 m^2^ g^−1^), together with enhancement light absorption due to the multiple reflections. When utilize water as electron sacrificial agent, hydrogen production is the main competitive reaction in the process of CO_2_ reduction.^[^ ^134^, ^135^ ^]^ In order to improve the selectivity of CO_2_ reduction under the existence of water, covering a carbon layer on the photocatalyst will make more protons to participate in CO_2_ reduction. In this case, Pan et al. ^[^ ^136^ ^]^ reported wrapped a 5 nm thick carbon layer outside the In_2_O_3_, exhibited photoactivity of CO and CH_4_ evolution rate of 126.6 and 27.9 µmol h^−1^, respectively. The greatly enhancement performance was attributed to the improved chemisorption of CO_2_, which increased the chance of proton capture by the CO_2_^−^. In addition, Organic porous polymers such as C_3_N_4_, polymer and BN have been also explored in recent years.^[^ ^137^, ^138^ ^]^ Yu and co‐workers^[^ ^139^ ^]^ synthesized hierarchical porous O‐doped g‐C_3_N_4_ by heating, exfoliating, and curling‐condensation of bulk g‐C_3_N_4_. The methanol evolution rate is 0.88 µmol g^−1^ h^−1^, fivefold higher than that of bulk g‐C_3_N_4_ (0.17 µmol g^−1^ h^−1^). The greatly enhanced photoactivity was mainly resulted from the porous g‐C_3_N_4_ with higher specific surface area, enhanced light absorption, together with more exposed active edges. This work paves a novel way to design hierarchical porous nanostructures. However, the photocatalytic evolution rate needs to be improved.

Inorganic porous semiconductor ZnO was investigated by Long and co‐workers^[^ ^140^ ^]^ ZnO forms 3D holes at high temperature, so that metal particles can be fixed to ZnO in the subsequent synthesis process, and this can make the metal particles evenly distributed. Utilization of the surface plasmon resonance (SPR) of noble metals, the as‐prepared samples exhibited high yield of CH_4_ and CO. Similarly, He and co‐workers investigated the effect of defects in porous ZnO nanoplate on CO_2_ photoreduction.^[^ ^141^ ^]^ In this work, defects in the porous ZnO accelerate separation of photogenerated carriers, resulting in greatly enhanced photocatalytic performance. However, the stability and photoactivity of the photocatalyst are desirable to improve. Furthermore, intermediate species for the CO_2_ photoreduction on defective ZnO should be deep investigated. Chromium (Cr) doped mesoporous CeO_2_ was synthesized via a nanocasting route.^[^ ^142^ ^]^ The mesoporous structure could enhance the uptake capacity of CO_2_, leading to high photocatalytic activity. However, it is still a challenge to synthesize porous inorganic semiconductors directly due to its inflexible tunability in structure. In the past few years, many porous semiconductors were derived from heating MOFs due to its intrinsic porous structure.^[^ ^143^, ^144^, ^145^ ^]^ Li and co‐workers first reported the porous hierarchical TiO_2_ derived from MIL‐125(Ti) for photocatalytic CO_2_ reduction.^[^ ^146^ ^]^ TiO_2_ with high surface area modified with basic MgO, leading to high photocatalytic CO_2_ reduction activity. Lou et al. synthesized a series of compounds with hierarchical structure such as In_2_S_3_‐CdIn_2_S_4_ (Figure 12c,d) and sandwich‐like ZnIn_2_S_4_‐In_2_O_3_ based on MIL‐68 as precursor. The hierarchical structure is benefit to light absorption through light scatting and reflection, reduce the free path of carrier diffusion, increase the reaction contact area with CO_2_, thus greatly improves the efficiency of CO_2_ photoreduction.^[^ ^147^, ^148^ ^]^ Wang et al.^[^ ^149^ ^]^ synthesized porous polymer‐TiO_2_‐graphene (HCP‐TiO_2_‐FG) photocatalyst for the conversion of CO_2_ under visible light irradiation (Figure 12e). This composite, with large surface area 988 m^2^ g^−1^ and CO_2_ uptake capacity due to its porous structure, exhibited high CH_4_ formation rate of 27.62 µmol g^−1^ h^−1^ without any sacrificial reagents or co‐catalysts. This work provided a prototype of the combination of microporous organic polymers used in CO_2_ photoreduction.

### 4.4 Comparison between Four Kinds of Porous Materials

**Scheme 3:**
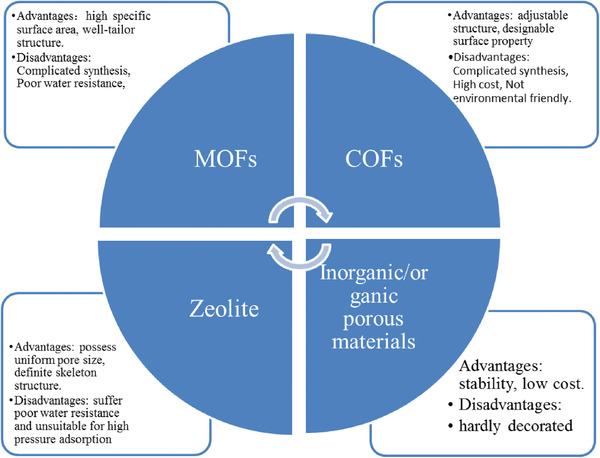
The advantages and disadvantages of four kinds of porous materials.

Photocatalyst with high CO_2_ uptake is a necessary condition for catalytic CO_2_ conversion. Porous materials such as MOFs, COFs, molecule sieve and inorganic/organic porous materials have attracted considerable attentions in the CO_2_ conversion field due to their high specific surface area and well‐tailor structure.^[^ ^25^ ^]^ However, each kind of porous material has its advantages and disadvantages. For instance, MOFs exhibited large surface area and flexible tunability structure compared with inorganic porous materials but suffered poor water resistance. On the other hand, the inorganic porous material is so hardly decorated that it so difficult to study the structure–activity. With regard to the molecule sieves, they possess uniform pore size, definite skeleton structure. However, just as MOFs, molecule sieves also suffer poor water resistance and unsuitable for high pressure adsorption. In recent years, COFs have attracted considerable attentions because of adjustable structure. However, the synthesis method is too complex and involves many organic compounds compared with inorganic porous material, which is not environmentally friendly. The advantages and disadvantages of different porous materials are summarized in **Scheme** **3**.

## 5 Conclusions and Perspectives

The development of porous materials with high catalytic efficiency is an important research area given their diverse chemical structures and multitudinous applications (**Table** **2**). This progress report summarizes the recent advances in MOFs‐based materials for photocatalytic CO_2_ reduction from three critical photocatalytic aspects, say light absorption, carrier dynamics, and relationship between CO_2_ uptake capacity and activity. Owing to their intrinsic porosity and smooth implementation of function moieties, the catalytic performance can be improved fundamentally. As highlighted above, porous materials can be used as solid state photocatalysts that will harvest visible light and provide active catalytic centers simultaneously within a single structure. Their CO_2_ reduction performance can be enhanced by either tuning the building blocks for more efficient light absorber, adjusting the metal centers to improve adsorption capacity and catalytic activity, or a conjunction of both. In addition, a large number of porous materials allow them to couple with molecular catalysts, photosensitizer molecules, semiconductors or plasmonic metal clusters, thereby yielding novel, high surface area composite photocatalysts to present a higher catalytic activity. In particular, most porous materials have potential for CO_2_ capture capacities, which may promote their applications at low CO_2_ concentration conditions. Above all, porous materials hold their unique advantages in solar‐driven CO_2_ reduction and all the synthetic strategies will offer the rational design of porous material‐based photocatalysts with excellent catalytic performance.

**Table 2:** Porous material‐based for photocatalytic CO_2_ reduction. TEOA: triethanolamine, TEA: triethanolamine, TON: turnover number

Photocatalysts	Reaction medium	Major product	Max rate evolution	Ref.
NH_2_‐MIL‐125(Ti)	MeCN/TEOA (5:1)	HCOO^−^	8.14 µmol, 10 h	^[^ ^58^ ^]^
NH_2_‐UiO‐66(Zr)	MeCN/TEOA (5:1)	HCOO^−^	13.2 µmol, 10 h	^[^ ^59^ ^]^
Zr‐SDCA‐NH_2_	MeCN/TEOA (30:1)	HCOOH	96.2 µmol h^−1^ mmol _MOF_^−1^	^[^ ^61^ ^]^
Zn/PMOF	Water (vapor)	CH_4_	8.7 µmol h^−1^ g^−1^	^[^ ^66^ ^]^
NNU‐28	MeCN/TEOA (30:1)	HCOOH	52.8 µmol g^−1^ h^−1^	^[^ ^67^ ^]^
Al‐PMOF	100 mL water + 1 mL TEA	CH_3_OH	262.6 ppm g^−1^ h^−1^	^[^ ^68^ ^]^
UiO‐67/Re^I^(dcbpy) (CO)_3_Cl	MeCN/TEA = 20:1	CO	TON = 10.9	^[^ ^71^ ^]^
Hf_12_‐Ru‐Re/[Ru(bpy)_3_]^2+^	1.9 mL CH_3_CN + 0.1 mL TEOA	CO	TON = 3849	^[^ ^72^ ^]^
NH_2_‐UiO‐66(Zr/Ti)	MeCN/TEOA (5:1)	HCOO^−^	5.8 mmol mol^−1^	^[^ ^75^ ^]^
UiO‐66‐CrCAT	MeCN/TEOA (4:1)	HCOOH	51.73 µmol, 6 h	^76]^
ZIF‐8/Zn_2_GeO_4_	Na_2_SO_3_	CH_3_OH	2.44 µmol g^−1^	^[^ ^80^ ^]^
Co‐ZIF/g‐C_3_N_4_	MeCN:H_2_O = 3 : 2 TEOA = 1 mL	CO	20.8 µmol CO, 2 h	^[^ ^81^ ^]^
Co‐ZIF‐9/CdS	MeCN:H_2_O = 3 : 2 TEOA = 1 mL	CO	85.6 µmol, 3 h	^[^ ^82^ ^]^
Co‐ZIF‐9/TiO_2_	3 mL water	CO	8.79 µmol, 10 h	^[^ ^83^ ^]^
Cu_3_(BTC)_2_@TiO_2_	Water (vapor)	CH_4_	2.64 µmol g^−1^ h^−1^	^[^ ^84^ ^]^
NH_2_‐UiO‐66/TiO_2_	CO_2_/H_2_ (1.5 v/v ratio)	CO	About 5 µmol g^−1^ h^−1^	^[^ ^85^ ^]^
PCN‐222	MeCN/TEOA (10:1)	HCOO^−^	30 µmol, 10 h	^[^ ^86^ ^]^
MAPbI_3_@PCN‐221(Fe_0.2_)	MeCN/TEOA (v/v, 1:0.012)	CH_4_	1028.94 µmol g^−1^	^[^ ^87^ ^]^
Cd_0.2_Zn_0.8_S@UiO‐66‐NH_2_	100 mL 0.1 m NaOH	CH_3_OH	6.8 µmol h^−1^ g^−1^	^[^ ^90^ ^]^
Ag⊂Re_3_‐MOF‐16 nm	MeCN/TEOA (20:1)	CO	TON ≈ 0.1	^[^ ^92^ ^]^
Ag@Co‐ZIF‐9	MeCN/TEOA/H_2_O = 4:1:1 (v/v)	CO	28.4 µmol, 0.5 h	^[^ ^93^ ^]^
Pt/NH_2_‐MIL‐125(Ti)	MeCN/TEOA (5:1)	HCOOH	12.96 µmol, 8 h	^[^ ^96^ ^]^
MOF‐525‐Co	MeCN/TEOA (4:1)	CO	200.6 µmol g^−1^ h^−1^	^[^ ^99^ ^]^
Ni MOLs	MeCN/TEOA/H_2_O = 3:1:2 (v/v)	CO	12.5 µmol h^−1^	^[^ ^39^ ^]^
DA‐CTF	MeCN/TEOA (2:1)	CO	9.3 µmol, 2 h	^[^ ^112^ ^]^
TAPBB‐COF	1 mL Water	CO	295.2 µmol g^−1^	^[^ ^113^ ^]^
DQTP COF‐Co/Zn	MeCN/TEOA = 4:1	CO	1.020 µmol h^−1^ g^−1^	^[^ ^114^ ^]^
TTCOF‐Zn	Water	CO	12.33 µmol, 60 h	^[^ ^115^ ^]^
ex‐Ti‐oxide/Y‐zeolite	Water	CH_4_	10 µmol g^−1^ h^−1^	^[^ ^121^ ^]^
Pt‐loadedTi‐MCM‐48	Water	CH_4_	12 µmol g^−1^ h^−1^	^[^ ^121^ ^]^
MgO/Ti‐MCM‐41	Water	CH_4_	157 ppm g^−1^ h^−1^	^[^ ^125^ ^]^
HZSM‐5	Water	CO	3.32 µmol g^−1^ h^−1^	^[^ ^126^ ^]^
g‐C_3_N_4_ nanotubes	MeCN/TEOA/H_2_O = 3:1:2 (v/v)	CO	103.6 µmol g^−1^ h^−1^	^[^ ^127^ ^]^
CdS@BPC‐700	MeCN/TEOA/H_2_O = 3:1:1 (v/v)	CO	39.3 µmol g^−1^ h^−1^	^[^ ^128^ ^]^
CdS/Mn_2_O_3_	Water (vapor)	HCOH	1392.3 µmol g^−1^ h^−1^	^[^ ^129^ ^]^
Au/m‐ZnO‐4.6	Water	C_2_H_6_	27 µmol g^−1^ h^−1^	^[^ ^140^ ^]^
ZnIn_2_S_4_–In_2_O_3_	MeCN/TEOA/H_2_O = 3:1:2 (v/v)	CO	3075 µmol g^−1^ h^−1^	^[^ ^148^ ^]^

Although great progress has been made, challenges remain toward commercialization of porous materials for solar‐driven CO_2_ reduction.

1) Carrier dynamics includes carrier separation, lifetime, mobility, average diffusion free path, and other important factors. However, in terms of porous materials, rare work focuses on two key kinetic parameters, i.e., carrier mobility and average diffusion length. These two kinetic parameters determine the effective utilization of photogenerated charge, and then dominate the photocatalytic reaction rate.
2) Few studies were reported on the electron transfer mechanism between various ligands and metal clusters. More research is required to consider the band structure of different ligands and combine with high‐throughput theoretical calculation to design porous photocatalytic materials with high carrier separation and fast mobility.
3) Regarding porous materials, there are few studies on the selectivity of photocatalytic reduction of CO_2_. Especially on the effect of pore size or pore volume on the activity and selectivity remains to be discussed. It is anticipated that there will be new insights of the selectivity of porous materials in the future.
4) At the present, there is a lot of research on the membrane formation of porous materials, such as the separation of gas by MOFs. Therefore, the membrane‐forming characteristics of MOF can be used for catalytic reaction, which is conducive to the subsequent separation steps and can save a lot of separation cost.
5) Finally, most of the studies are still in the case of pure CO_2_ and the need for electronic sacrificial agent or photosensitizer. Reaction with low concentration of CO_2_ and water is more suitable for industrialization, energy saving and emission reduction. In addition, at present, the technology of photocatalytic CO_2_ reaction device is still at the most basic stage, and more reaction device design is needed to further promote the industrialization process of photocatalytic CO_2_.

In conclusion, it remains far from optimal performance of porous materials for solar‐driven CO_2_ reduction. This progress report is expected to offer new viewpoints to promote the development of highly efficient CO_2_ photoreduction systems.

## Conflict of Interest

The authors declare no conflict of interest.
